# Chronic Follicular Prostatitis

**Published:** 1898-09

**Authors:** Charles Ferdinand Durand

**Affiliations:** Buffalo, N. Y.


### CHRONIC FOLLICULAR PROSTATITIS.

By CHARLES FERDINAND DURAND, B. A., M. B., Buffalo, N. Y.

THE term prostatitis is often loosely used to denote several different pathological conditions, each of which is characterised by more or less distinctive signs and symptoms. This is the more to be regretted as the treatment beneficial in one of these morbid states may be useless or injurious in another. For this reason the various diseased processes peculiar to the posterior urethra, seminal vesicles and prostate should be carefully differentiated from each other so that appropriate treatment may be intelligently instituted.

A recent work on genito-urinary diseases treats chronic prostatitis as an entity and states that its symptoms are practically those of posterior urethritis, including frequent and painful urination, deep perineal pain, which radiates to the rectum and scrotum and down the thighs, painful defecation, loss of sexual desire and frequent pollutions.

Now here we have a great variety of symptoms, not one of which is necessarily present in chronic inflammation of the follicles of the prostate. It is true that associated with the follicular inflammation we very often find inflammation of the posterior urethra, ejaculatory ducts, seminal vesicles and ampullæ and frequently a parenchymatous inflammation of the gland, but on the other hand cases of chronic inflammation of the follicles occur uncomplicated by these other morbid conditions. When frequent micturition occurs it indicates not follicular prostatitis, but an irritation of the mucous membrane of the posterior urethra due to inflammation, acid urine or some reflex; pain deep in the perineum, radiating to rectum, scrotum and thighs, points to a parenchymatous inflammation of the prostate, when vesical calculus is excluded; pain after defecation also indicates a parenchymatous inflammation; loss of sexual power and frequent pollutions, show that there is disease of the sexual nervous arc, either at center or periphery.

Chronic follicular prostatitis is usually the result of a septic inflammation of the posterior urethra of gonorrheal origin and therefore the signs and symptoms of inflammation of this part of the urethral canal and contiguous structures are often present.

What I wish to point out, however, is that the inflammation may become localised, being confined to the follicles of the prostate and give rise to no symptoms whatever. In these cases the only sign that anything is wrong may be the presence in the urine of prostatic secretion, consisting of epithelial and pus cells, granular phosphates and occasional gonococci, together with more or less mucus. On rectal palpation the prostate may feel normal, but on being subjected to a milking process the contents of the follicles can be expressed. When in sufficient quantity these contents may appear at the meatus subsequent to the massage, or when less in amount they will be found in the urine. The great danger of this class of cases lies in the fact that from the absence of symptoms frequent coitus is indulged in and the gonorrheal disease communicated to others.

As to treatment, deep instillations of nitrate of silver, so frequently beneficial in chronic inflammation of the mucous membrane of the posterior urethra, are here of little or no value, because the solution does not enter the diseased follicles. The treatment that as given the best results in my hands, is massage of the prostate through the rectum and the use of the prostatic dilator and psychrophore. By stretching the prostatic urethra the follicles are emptied of more or less of their contents and the circulation through the gland is improved. The psychrophore, used with cold water, seems to improve the tone and aid in restoring the mucous membrane of the follicles to a healthy condition. Massage through the rectum not only empties the follicles most effectually, but promotes the decongestion and nutrition of the gland.

In employing massage I have found it advantageous to press with the finger first in an upward direction, especially at the lateral borders, of the prostate in order to empty the veins of the prostatic plexus and afterward direct the pressure toward the apex of the gland to empty the follicles. I frequently follow the massage treatment with a jet of cold water, directed against the under surface of the prostate through the rectum. These cases often try the patience of both surgeon and patient, but the treatment outlined above usually proves curative if persisted in.

